# Potential of Electrochemical Charge Injection for Quantum Dot Light-Emitting Devices

**DOI:** 10.1021/acs.chemmater.5c00579

**Published:** 2025-06-02

**Authors:** Hua Chen, Reinout F. Ubbink, Rens A. Olsthoorn, Maarten Stam, Jesse ‘t Hoen, Tom J. Savenije, Arjan J. Houtepen

**Affiliations:** Optoelectronic Materials Section, Faculty of Applied Sciences, 2860 Delft University of Technology, van der Maasweg 9, Delft 2629 HZ, The Netherlands

## Abstract

The efficiency of quantum dot (QD) light-emitting diodes is limited by inefficient hole injection into the valence levels of the QDs. Electrochemical doping, where mobile ions form electrical double layers (EDLs) at electrodes, offers a route to removing injection barriers. While QD light-emitting electrochemical cells (QLECs) have shown promise, prior studies relied on additional charge injection layers, complicating the study of charge injection into QDs. In this work, devices with a simple ITO/QD active layer/Al structure were fabricated using highly photoluminescent ligand-exchanged CdSe/CdS/ZnS QDs, poly­(ethylene oxide), and lithium trifluoromethanesulfonate as electrolyte. We show that the dense QD films in these QLECs can be electrochemically doped, transport charges, and exhibit electroluminescence. Symmetrical cyclic voltammograms and operando photoluminescence measurements prove that these devices function as electrochemically doped LECs. Spectroelectrochemical experiments on separately n- and p-doped QD films indicate that hole injection remains the primary limitation in QLEC performance. These findings demonstrate that using EDLs to facilitate charge injection in QD light-emitting devices is promising, but significant challenges remain to be solved before electron and hole injections are balanced.

## Graphical abstract

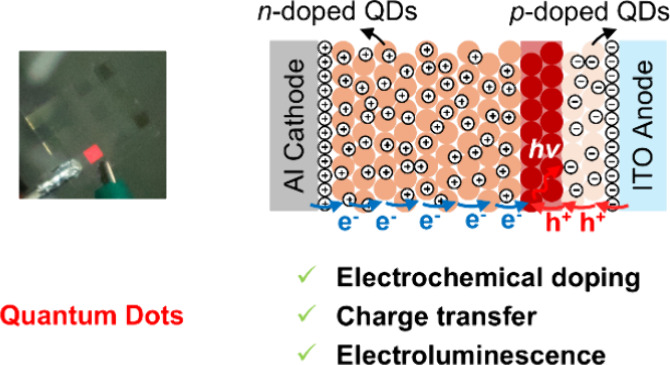

## Introduction

Quantum Dots (QD) are increasingly considered as emitting materials in lighting applications. The external quantum efficiency (EQE) of QD light-emitting diodes (QLEDs) has increased from ∼0.01% in the earliest report by Alivisatos et al. from 1994 to over 30% recently.^1–3^ The EQE of QLEDs is invariably found to be limited by inefficient hole injection, typically attributed to the deep-lying valence levels of CdSe and InP QDs and the resulting energy offset with the work function of the anode. The most efficient QLEDs hence use advanced structures of electron and hole injection layers to facilitate hole injection and balance electron and hole injection.^4–6^ However, even in these cases, it is shown that electrons are easily injected, while hole injection is inefficient. Electroluminescence results from the slow injection of holes into negatively charged QDs.^7^

An alternative method to inject charge carriers is by using electrochemistry. In this case an electrical double layer (EDL) of a charged electrode and mobile counterions provides an interface potential drop that facilitates electron or hole injection and eliminates the energy offset between the work function of the electrode and the conduction or valence levels of the QDs.^8,9^ This concept of electrochemical electron and hole injection has been leveraged in light-emitting electrochemical cells (LECs).^10,11^

LECs consist of two electrodes sandwiching a single active layer, which contains electroluminescent materials mixed with mobile ions. As illustrated in Scheme 1, because of the mobile ions present in the LEC active layer, EDLs form at the interfaces between the metallic electrodes and the active layer, which eliminates the energy barriers for injection of electrons and holes into the active layer. When charges are injected into the electroluminescent material, the additional charges are compensated by mobile ions throughout the active layer. This *in situ* electrochemical doping results in the formation of n- and p-type regions. A p-i-n junction is formed in the middle of the device, where the injected carriers radiatively recombine to obtain electroluminescence (EL).^12,13^ The EDLs and electrochemical doping eliminate the need for complex multilayer structures.^14–16^ The simplicity of this device structure can lead to reduced manufacturing costs and improved device scalability. Furthermore, there are, in principle, no inherent restrictions on the electrode materials of LECs, since the potential drop in the EDL eliminates injection barriers between the electrodes and the active material, offering opportunities to implement air-stable electrodes.

**Scheme 1:**
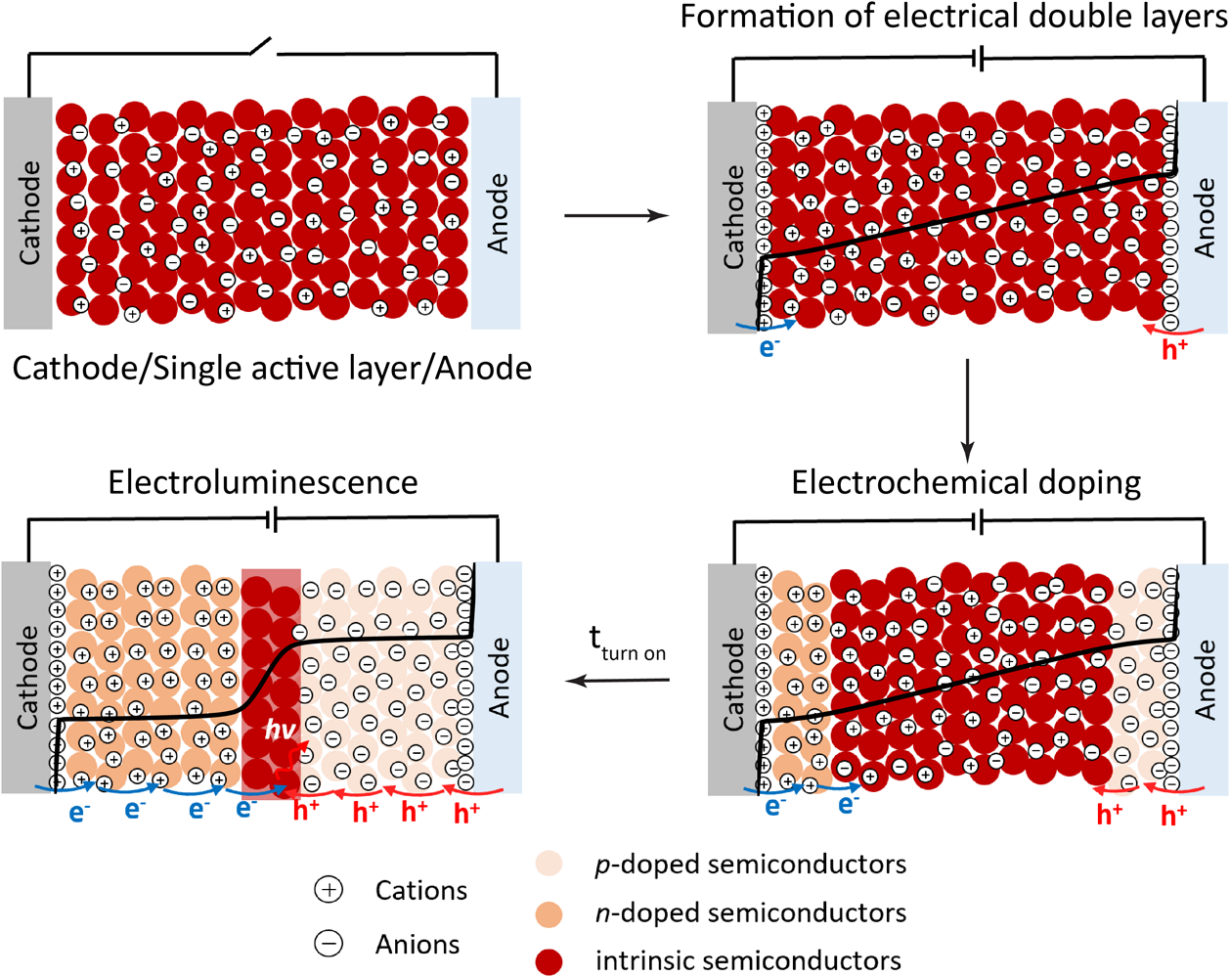
Working Mechanism of LECs, Illustrating the Formation of EDLs, Electrochemical Doping, and EL^a^

a The potential distribution across the active layer is shown in black solid lines. EDLs are first formed by the accumulation of mobile ions at the electrodes. This reduces the injection barrier of charges, which are subsequently injected into the material. The additional charge is compensated by the flow of oppositely charged ions, resulting in net n- and p-type doped regions. These doped regions facilitate charge transport to the intrinsic region in the middle of the device, where holes and electrons recombine to emit light

Polymers and ionic transition metal complexes (iTMC) have been the most widely chosen electroluminescent materials in LECs.^17–20^ However, relatively low photoluminescence quantum yield (PLQY), difficulty in tuning emission colors, and poor stability impede the commercialization of LECs.^21–23^ Colloidal quantum dots (QDs) offer opportunities to address the aforementioned issues due to their size-dependent emission spectra, near-unity PLQY, and high color purity for light-emitting applications.^24–27^

Robust inorganic QDs have been implemented into polymer and iTMC-based LECs, acting as color-supplementing materials.^28,29^ With careful device structure design to balance the carrier injection, pure QD electroluminescence and efficient device performance can be obtained.^30,31^ However, with iTMC as the charge injection layer, these hybrid devices still suffer from the traditional degradation issues of LECs and the multilayer structure is complicated to fabricate. To overcome this issue, there have been attempts to fabricate LECs using CdSe/CdS, InP/ZnSeS, and CuInS_2_/ZnS QDs as the only electroluminescent materials in the active layer, by blending these QDs with poly-(*N*-vinylcarbazole) (PVK) and ionic liquid as charge transporting matrix.^32–34^ PVK is widely used as a host to transport charges to the electroluminescent guest, which complicates the charge transfer.^35,36^ Emission from PVK also broadens the emission spectrum.^34^ The additional charge transport materials result in extra voltage losses, since the PVK band gap is much larger than that of the emitting QDs.

More importantly, the working mechanism of QD-based LECs (QLECs) remains elusive due to the additional charge transport materials and injection layers used. *In situ* electrochemical doping of QDs, which facilitates charge transport and makes QLECs different from other light-emitting devices, has not been experimentally illustrated so far. At the same time, it is known that films of QDs can be electrochemically doped,^37–40^ that the doped QD films can transport charge efficiently if short-enough ligands are used^41–47^ and that they can exhibit efficient EL. In principle, dense QD films could play all roles needed in an LEC, and could be used as a single active layer in an LEC. However, until now, no EL has been obtained from QLECs where no additional charge injection or transport materials were employed. This allows a simpler device architecture and, more importantly, allows us to probe the injection of electrons and holes and the formation of doped regions more directly, providing insight into the working mechanism of QLECs and the potential of using EDLs to lower the injection barrier for holes in QD light-emitting devices in general.

In this work, we fabricated QLECs with a simple device structure of an ITO/QD active layer/Al, without adding charge injection and transport layers. In the active layer, highly photoluminescent ligand-exchanged CdSe/CdS/ZnS QDs were used, while lithium trifluoromethanesulfonate (LiCF_3_SO_3_) in poly­(ethylene oxide) (PEO) was used as electrolyte. These devices reproducibly show EL from the QDs. Cyclic voltammetry and operando PL measurements were used to investigate the working mechanism of our devices. Reversible PL quenching during operation demonstrates that electrochemical doping takes place, which is typical of LECs. We compare the experimental characteristics to drift-diffusion simulations and conclude that our devices operate as LECs rather than as diodes.

Although our devices can be charged from both sides, no EL is obtained when Al is the anode. This suggests that the intrinsic recombination zone occurs close to the anode, quenching the EL if the anode is metallic Al. The asymmetric p-i-n junction is likely due to the fact that, in spite of the use of EDLs, hole injection remains inefficient. To gain further understanding of charge injection and transport, we performed spectroelectrochemical (SEC) measurements on the QD films deposited on an ITO electrode. The results indicate that electrons are readily injected into QDs to form the n-type region in QLECs, while there is no clear spectroscopic evidence of hole injection. We discuss the possible reasons why hole injection remains inefficient even when EDLs are used, which should eliminate the injection barriers. These observations show that the use of EDLs to facilitate charge injection in QD light-emitting devices is promising, but significant challenges remain to be solved before electron and hole injections are balanced.

## Results/Discussion

Highly photoluminescent red-emitting core/shell/shell QDs were synthesized by sequential shelling of approximately 6 monolayers of CdS and 2 monolayers of ZnS on CdSe QDs (see the Supporting Information for details). The thin ZnS shell is essential to achieve reversible electron injection by removing trap states localized on the CdS surface and enhancing the electrochemical stability of the QDs against cathodic degradation.^48^ During the shelling, sulfur-octadecene (S-ODE) was used as a sulfur source instead of thiols to have only oleate ligands on the surface of QDs, simplifying the ensuing ligand exchange procedure. The photoluminescence quantum yield (PLQY) of the as-synthesized QDs is 87% with an emission peak maximum at 649 nm (Figure 1a). The broadened peak in the NMR spectrum of the as-synthesized QDs, observed at 5.3–5.4 ppm, is typical for the protons of the double bond in bound oleate ligands (Figure 1b).^49^ The X-ray diffractogram of QDs demonstrates a zinc blende crystal structure (Figure 1c). TEM images reveal that QDs are octahedral with a size of around 10 nm (Figure 1d).

**Figure 1:**
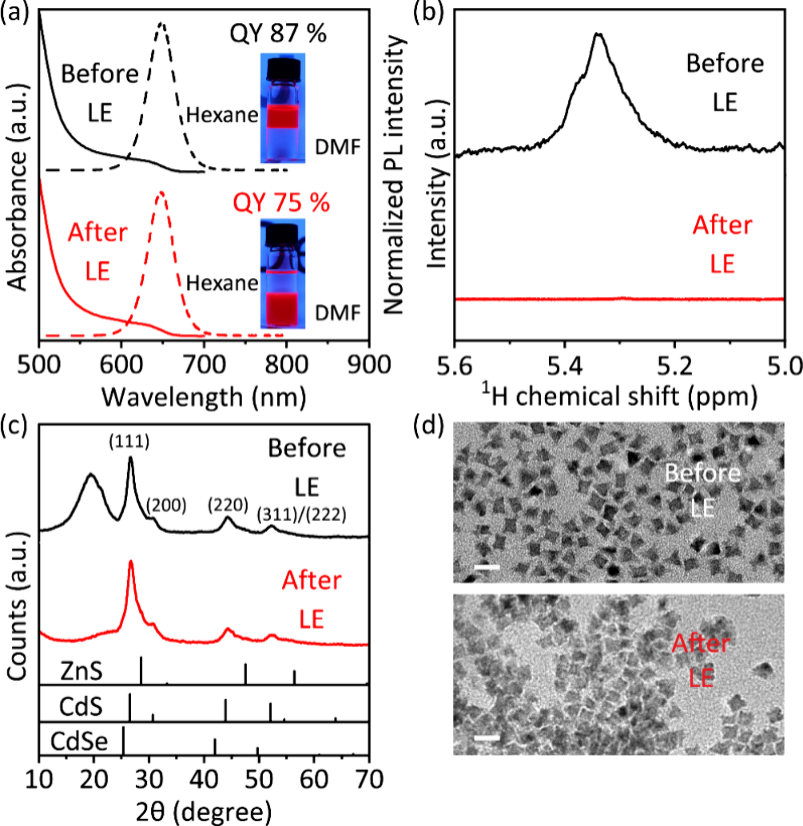
(a) Absorption (solid lines) and PL (dash lines) spectra, (b) ^1^H NMR spectra, (c) X-ray diffraction patterns, and (d) TEM images of CdSe/CdS/ZnS QDs before and after ligand exchange. Inset in (a) photographs of QDs transferred from hexane (top) to DMF (bottom) after a two-phase ligand exchange. The standard PDF cards of CdSe, CdS, and ZnS in (c) are 19–0191, 75–1546, and 01–0792, respectively. Scale bars in (d) 20 nm.

To realize efficient charge transfer between QDs in LECs, the long-chain insulating ligands on the surfaces of the as-synthesized QDs must be removed or replaced by shorter ligands. A ligand exchange (LE) is also necessary to transfer QDs into polar solvents to ensure miscibility of the QDs and electrolyte. However, LE often results in the formation of new traps on the surface of the QDs and therefore a drop in PLQY.^50–54^ To maintain the PLQY after LE, we used In­(NO_3_)_3_ to perform the LE, a method recently developed by Talapin et al.^55^ The original oleate ligands are removed, leaving a positively charged QD surface, which together with loosely bound NO_3_^–^ ions ensures stable dispersion in a polar system, as demonstrated by a two-phase LE, where QDs are transferred from hexane (top phase) to *N*,*N*-dimethylformamide (DMF) (bottom phase) (inset of Figure 1a).

The stripping of oleate ligands was confirmed by ^1^H NMR spectroscopy. After LE, the peak of bound oleate is hardly observed, indicating the complete removal of oleate ligands (Figure 1b). The absorption and PL spectra remain the same after LE, while the PLQY is only marginally reduced to 75% (Figure 1a). The full width at half-maximum (fwhm) of the PL spectra is 36 nm before and after the treatment, indicating an unchanged narrow size distribution and high color purity.

The X-ray diffractogram reveals no changes in crystal structure after LE. The peak located at around 19° is assigned to the ordered oleate ligands on QDs, which disappears after the ligand exchange, indicating the removal of organic ligands (Figure 1c).^56^ As shown in the TEM images, the morphology of QDs is retained, while the distance between QDs is decreased due to the removal of long-chain ligands (Figure 1d). In conclusion, after LE, QDs are highly photoluminescent and form stable colloidal dispersions in DMF, after which they are ready for the fabrication of QLECs.

QLECs with a device structure of the ITO/QD active layer/Al were fabricated (Figure 2a). In the active layer, ligand-exchanged QDs were combined with PEO and LiCF_3_SO_3_ ions. The active layer was directly spin-coated onto an ITO/glass substrate. The Al back contact with a thickness of 100 nm was thermally evaporated onto the QD active layer (see Experimental Section for details). EL was obtained from this simple three-layer QLEC, as shown in the inset of Figure 2b.

**Figure 2:**
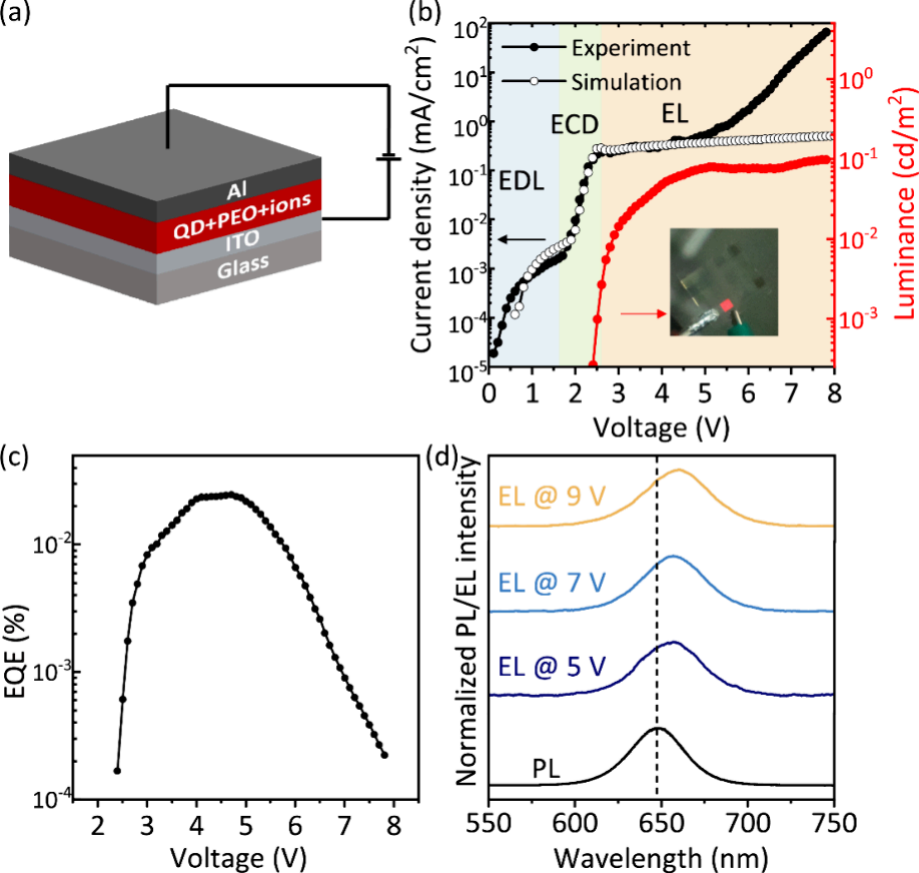
(a) Device structure of the QLECS: ITO/QD active layer/Al. (b) Experimental and simulated current density–voltage-luminance curves. EDL, ECD and EL denote electrical double layer, electrochemical doping and electroluminescence, respectively. Inset: photograph of a red-emitting QLEC in operation. (c) EQE and (d) normalized PL spectra of QDs dispersion and normalized EL spectra of QLECs at various voltages. ITO was biased positively in these measurements and the scan rate was 50 mV/s.

The current density–voltage–luminance characteristics of the red-emitting QLEC, with ITO as the positive electrode, can be divided into three stages: below 1.7 V, only a low background current is observed (first stage, marked in blue). The current density then increases exponentially between 1.7 and 2.7 V applied potential (second stage, marked green). Above 2.7 V, the current density is almost constant (third stage, marked orange) until starting to increase exponentially again at a potential higher than 5 V.

To understand this behavior, we have modeled the *J*–*V* response of the QLECs using drift-diffusion simulations, based on those by van Reenen et al.^57^ In these simulations, given a voltage program, the current response of the device is calculated based on drift-diffusion movement of electrons, holes, anions, and cations. Simulations were performed using any known parameters obtained from the experiments and then fitted to the experimental data to optimize the mobilities of the charge carriers for this specific case. Concentration profiles from the simulation (Figure S1) provide insight into the mechanisms underlying these three stages.

Below 1.7 V, the potential is not high enough to inject any carrier into the QDs. The only current is from the formation of the EDLs. When the potential is higher than 1.7 V, electrochemical doping starts to occur, which results in a significant increase in the current density over a short potential range. Above 2.7 V, the electrochemical doping process is completed and the current density levels off. At this point, the p-i-n junction is formed, and EL is observed due to charge recombination in the intrinsic zone. At 5 V, the luminance and the external quantum efficiency (EQE) reach the maxima of 0.1 cd/m^2^ and 0.024%, respectively (Figure 2b,c). In the simulations, the current density above 2.7 V increases only very slightly for higher applied potential due to a slight redistribution of the electrochemical doping at higher potentials (Figure S1). In the experimental device, current density is also nearly constant up to a potential of 5 V. Above 5 V, current density increases exponentially again while the luminance stays constant, resulting in a drop-off of the EQE. This implies leakage channels are opened by nonideal processes above 5 V.

When the device is scanned from 0 to 9 V, the EL spectra are 9–12 nm red-shifted compared to the PL spectrum. The fwhm of the EL spectra is increased to 45 nm (Figure 2d). In quantum dots light-emitting diodes (QLEDs), a red shift of the EL spectrum has been reported due to interdot interactions and electric-field-induced Stark effects.^58,59^ However, we also consider that electrochemical doping results in the preferential charging of the larger QDs in the ensemble, which subsequently emit slightly red-shifted compared to the PL, where all QDs are excited.

To investigate the working mechanism of the QLECs, we performed both chronoamperometry and cyclic voltammetry experiments. Figure 3a shows an experimental cyclic voltammogram (CV) of the QLECs, where a negative potential denotes that ITO is biased positively, while a positive potential denotes that ITO is biased negatively. Significant current density can be observed for both negative and positive applied bias, unlike in a diode. This symmetrical voltage dependence is also characteristic of LECs because the *in situ* p-i-n junction can be formed in both directions. When the device is scanned to a negative bias, the n-doped region is formed near the Al electrode, while the p-doped region is formed on the side of the ITO electrode. When the bias is scanned back to 0 V, the device returns to its intrinsic state as ions diffuse across the whole device. Then, when the bias becomes positive, the p-i-n junction is formed again with a reversed orientation.

**Figure 3:**
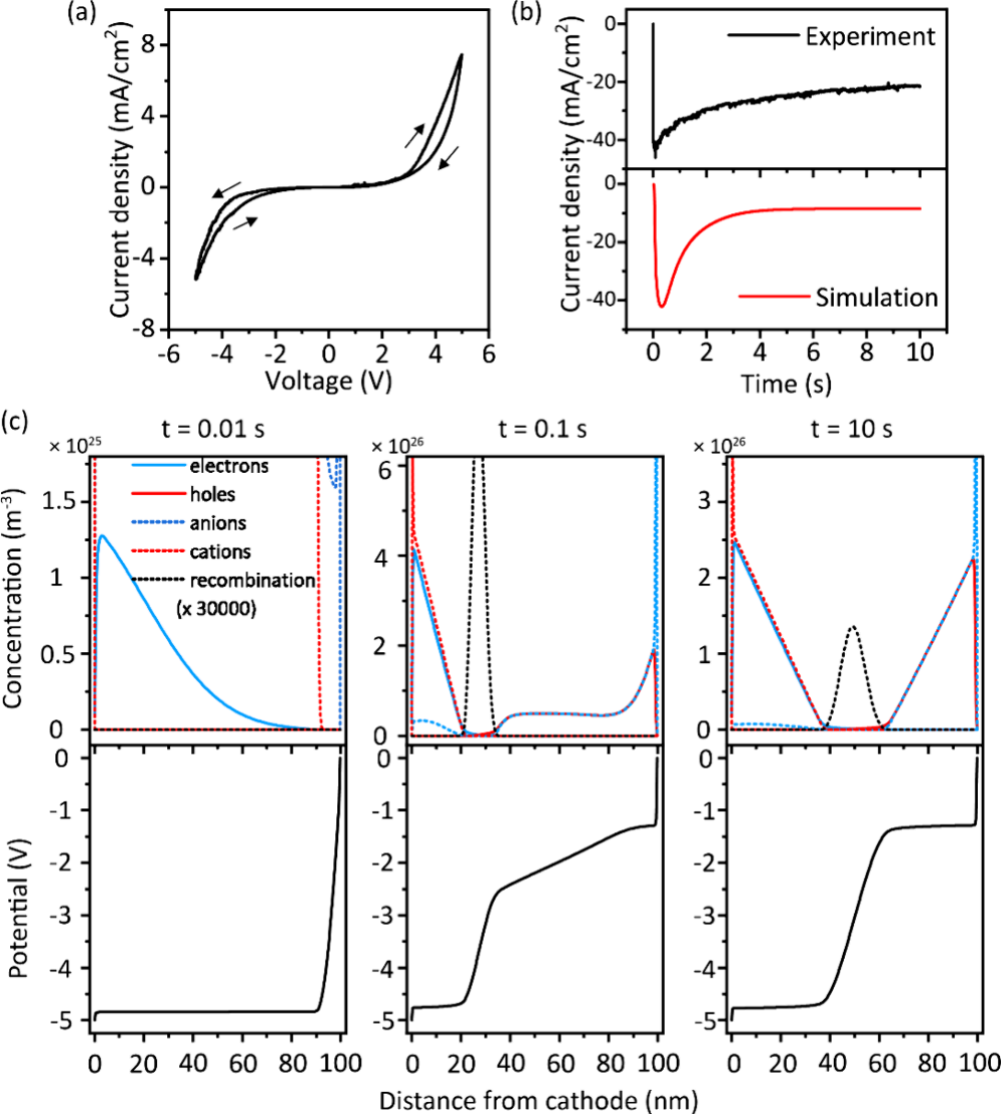
(a) Cyclic voltammogram of a QLEC. The scan rate was 50 mV/s. (b) Experimental and simulated electrical response of the device under a constant bias of −5 V. The ITO electrode was biased positively. (c) Simulated n-i-p junction evolution in an LEC under a constant bias of −5 V. Top panel: distribution of carriers, ions, and recombination concentrations from cathode to anode. Bottom panel: distribution of electrostatic potential from the cathode to anode.

The constant bias measurement, shown in the top panel of Figure 3b, shows three distinct parts. First, a quick increase in the current density is observed. Second, the current density drops, and finally, the current density reaches a steady-state value. This characteristic current response to constant applied bias (observable rise, peak, and steady state) indicates that the device undergoes electrochemical doping and is indeed working as an LEC. The simulated results of the constant bias response at −5 V, shown in the bottom panel of Figure 3b, show the same three contributions as observed in the experiment.

Snapshots of concentration profiles taken during the drift-diffusion simulations (Figure 3c) provide insight into the subsequent processes occurring during the constant bias experiment. Before electron and hole injection into the active layer occurs, EDLs first need to be formed next to the electrodes. However, even when ion mobilities are low, EDL formation takes place on time scales of <10 μs in the simulations, too fast to observe in the experiment. As soon as the potential drops in the EDLs are large enough to allow charge injection, the doping process starts. The speed of this process is limited by the mobility of the slowest charge carrier, which determines the response time of the device. The current density increases quickly, then reaches a maximum as the n and p doping fronts meet. After this, the doping process still continues, as the concentration profiles have not yet achieved the optimal charge distribution. During this period, increased electron–hole recombination is observed, since some drift transport of electrons and holes still occurs due to remaining electric fields, which results in a peak in the current density. Only after the doping process is fully completed and the steady-state doping profile is achieved does the current density reach a steady-state value.

In the steady-state situation, the current density is limited by the diffusion of electrons and holes in the doped zones. Since the current is constant over the device, this implies a constant gradient of the doping (to allow an equal diffusion current everywhere) and hence a linear concentration profile of ions, electrons, and holes. The position of the junction at steady state is dependent only on the ratio of electron/hole mobilities. The ion mobility determines the response time of the device but is irrelevant to the steady-state current. The concentration profile snapshots in Figure 3c show a simulated device where the anion mobility is much lower than the cation mobility, but this affects the junction position only while the doping process is incomplete. After completion of the doping process, the junction is positioned in the middle of the device since the electron and hole mobilities are equal in the simulation.

The occurrence and speed of in situ electrochemical doping in LECs can also be confirmed experimentally by analysis of the PL intensity as a function of the applied bias. Upon doping, the absorption of QDs is bleached and the PL is simultaneously quenched due to Auger recombination.^38,48,60^ To observe if electrochemical doping takes place in our devices, we therefore measured the PL of the QDs in a QLEC device in operation. In these measurements, a laser with a wavelength of 405 nm was used to illuminate the device from the glass substrate side, and the PL intensity was monitored by a fiber-coupled spectrometer, synchronized with the device operation (Figure 4a, Experimental Section). In this measurement, the device was driven by cyclic voltammetry.

**Figure 4:**
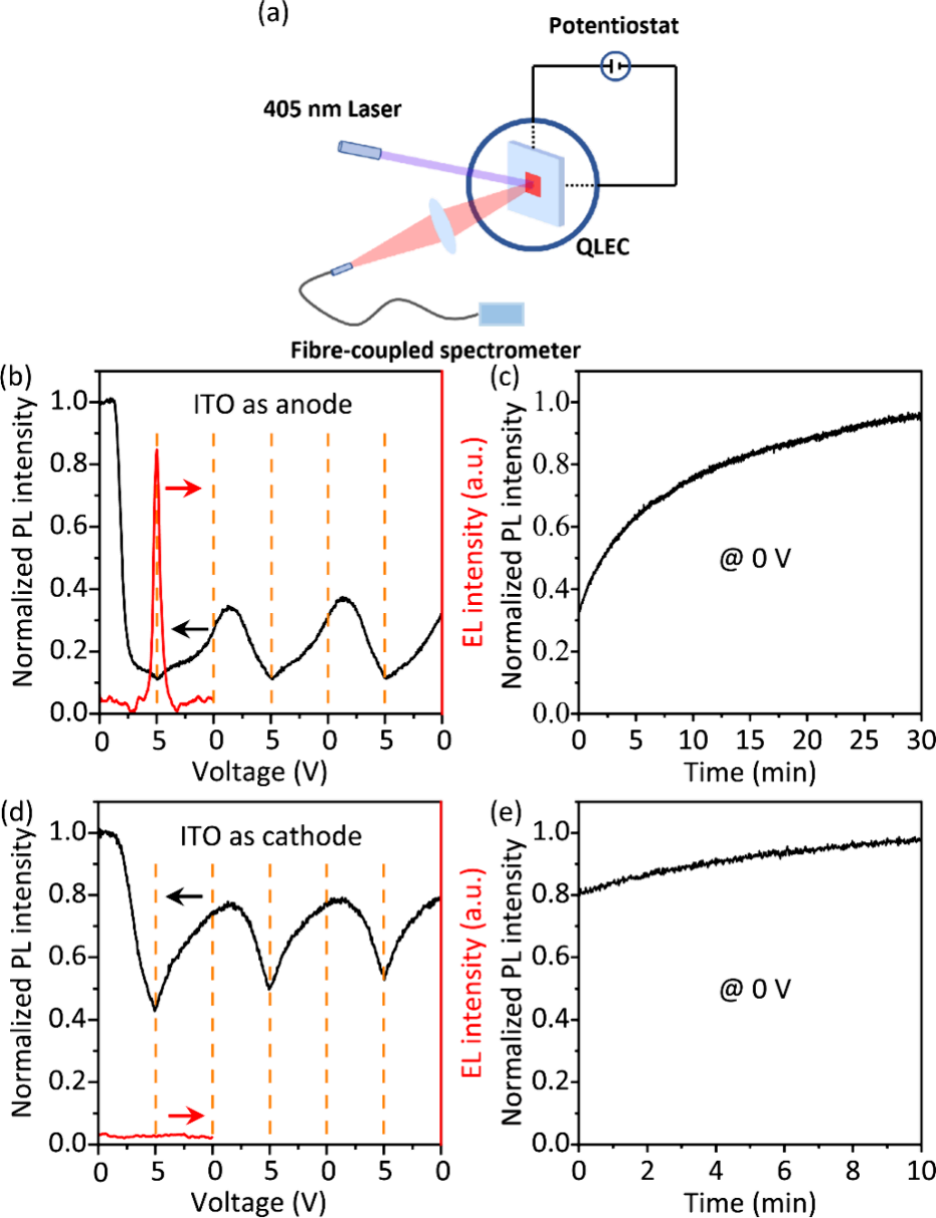
(a) Scheme of experimental setup for operando PL measurement. Operando PL (black solid lines) and EL (red solid lines) measurements were made when (b, c) ITO electrode was the positive anode and (d, e) ITO electrode was the negative cathode. During the measurement, the devices were electrically scanned from 0 to 5 V and back to 0 V for three cycles and then connected at 0 V for 10–30 min.

We carried out operando PL and EL measurements when the ITO electrode was biased positively. A typical EL evolution under cyclic potential sweep is shown in Figure 4b. EL starts around 2.5 V and increases with increasing potential up to a turning point of 5 V. PL was measured for 3 cycles. The onset of quenching in PL is around 1.3 V, and the PL intensity is decreased to 11% of the original intensity at 5 V. The quenching in PL can be divided into two regions, as indicated by the slope. From 1.3 to 2.5 V, the PL is rapidly quenched due to electrochemical doping. Beyond 2.5 V, the doping density does not increase much, and the injected carriers radiatively recombine in the emission zone. This clearly proves that QDs become electrochemically doped in the biased QLEC. When they are scanning back, the PL intensity is slowly recovered. After 3 cycles, the devices were biased at 0 V for 30 min. The PL intensity recovers to 96% of the original value, indicating that electrochemical doping is reversible (Figure 4c). The long recovery time can be ascribed to the slow emptying of charges in deep trap states or an overpotential of electrochemical reactions on the surface.^48^

In an ideal LEC, the device performance does not depend on the Fermi levels of the contacts since the EDLs can provide the required potential drop for electron and hole injection. There is, in principle, no limit to the magnitude of the potential drop in the EDLs; hence, any metal could be used as an anode or cathode. This also implies that the electrical and optical responses of LECs should be symmetric with respect to positive or negative biasing. In our devices, however, EL was not measurable when the ITO electrode was biased negatively (Figure 4d), even though significant current passed through the device as observed in Figure 3a. The electrochemical doping in this case was investigated by monitoring the PL intensity during operation. The onset of PL quenching is found at 1.6 V, which is larger than that when the ITO electrode is charged positively. The PL intensity is only quenched to 43% of the original intensity at 5 V in the first cycle (vs 11% PL left at 5 V when the ITO electrode is biased positively). The electrochemical doping process is similarly reversible, and 98% of the original PL intensity is recovered when the device is biased at 0 V for 10 min (Figure 4e). Apparently, the device does not respond fully symmetric to the electrical bias.

We hypothesize that because of difficulties with hole injection (see below), the emission zone is close to the anode. Therefore, when the ITO electrode is charged negatively, the emission zone is closer to the Al electrode. The emission of photons depends on the density of optical modes in the surroundings, which is much higher in the metal than in the active layer and the ITO electrode, as the dielectric constants of metals are infinite.^61,62^

In a QLEC device, the formation of both n- and p-type doped regions is crucial for device operation. Although the operando PL measurements illustrated in Figure 4 indicate electrochemical doping of the QDs, PL quenching resulting from electron doping and hole doping is hard to separate. To distinguish between electrochemical p- and n-type doping, we instead investigated the electrochemical doping process in QDs separately for electrons and holes using spectroelectrochemistry (SEC).^38,48,63^ Here, a three-electrode electrochemical cell is used where the working electrode and counter electrode (anode and cathode) are physically separated by the electrolyte solution. By manipulation of the Fermi level of the working electrode, either electrons or holes can be injected into the QD film.

For these SEC measurements, ligand-exchanged CdSe/CdS/ZnS QDs were drop-cast on ITO electrodes. To probe the electrochemical doping in our devices, we tried to use the same electrolyte environment for the SEC measurements. However, due to the high melting point of the PEO (Mv ≈ 5,000,000 g mol^–1^) used in the LEC device, it is not possible to perform the SEC measurements. Instead, we chose PEO with a smaller molecular weight of 600 g mol^–1^ as the solvent, ensuring chemical similarity while allowing liquid electrochemical measurements at room temperature. During cyclic voltammetry, a white light source and a laser with a wavelength of 405 nm were used to probe and excite the QD film alternately, and the corresponding absorption and PL spectra were collected by a spectrometer (Experimental Section, Figure S2). When a negative potential is applied to the working electrode, the absorption bleaching of the CdSe 1S transition (around 633 nm) and CdS localized transitions (around 500 nm) is observed, clearly indicating the electron injection into QDs (Figure 5b). A corresponding quenching in PL due to nonradiative Auger recombination is also present (Figure 5c). The absorption bleaching is reversible since it recovers when the potential is scanned back to the open circuit potential, demonstrating that the QDs are not degraded in three cycles (Figure 5d).

**Figure 5:**
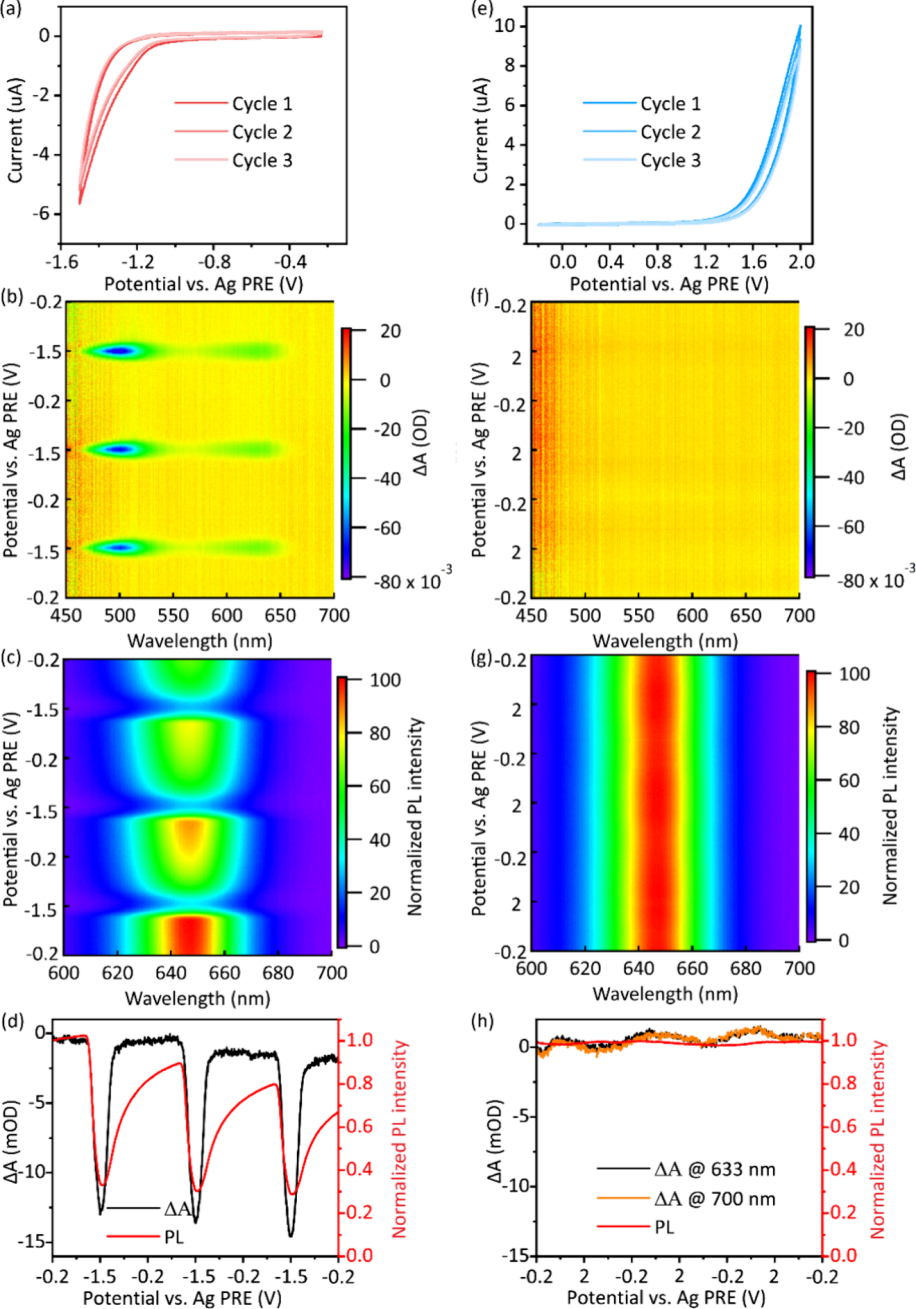
Cyclic voltammograms, 2D differential absorbance, 2D normalized PL and 1D plot of differential absorbance at the CdSe 1S transition and PL maxima of QD films/ITO scanned (a–d) negatively and (e–h) positively in the 0.1 M LiCF_3_SO_3_ PEO (Mn ≈ 600 g/mol) solution. The scan rate was 5 mV/s.

At positive potentials, no clear and reversible bleaching of the 1S absorption or PL quenching is observed (Figure 5f–h). There are some fluctuations in the differential absorption and PL spectra during the cyclic voltammetry. However, these do not correlate with the expected onset of hole injection (i.e., the valence band potential). Moreover, by comparison of differential absorbance at 633 nm (1S peak) and at 700 nm (inside the band gap), the absorption changes appear over the entire recorded spectrum, rather than showing a bleach of the band-edge absorption, as expected for hole injection (Figure 5h). Therefore, the changes in the spectra we observe here are not due to hole injection. Rather, we tentatively ascribe it to a change in the refractive index of the QD film in the electrolyte solution during charging.

We also performed the SEC measurements in 0.1 M LiClO_4_ acetonitrile electrolyte solution, which is one of the most widely used electrolyte solutions for electrochemical doping of QDs. As shown in Figure S3, electron injection under a cathodic current is clearly observed in absorption and PL spectra, but there is no clear spectroscopic indication of hole injection. It appears that the absence of hole injection does not depend on the electrolyte solution, but that it is the intrinsic property of CdSe/CdS/ZnS QDs.

The lack of hole injection in electrochemical doping experiments cannot be ascribed to the large energy offset between the electrode work function and the QD valence levels, since the EDL can, in principle, eliminate any injection barrier. Electrochemical experiments are routinely performed in very wide potential windows, for instance, in Li-ion batteries. While there is no intrinsic limit to the interface potential drop that can be generated, there could be competing processes that limit hole injection. For instance, electrochemical side reactions could occur that remove any injected holes, such as the oxidation of trace amounts of water or other impurities. This could explain the irreversible CVs in Figure 5e, which show that no injected holes are extracted on the reverse scan. The QDs themselves or species at their surface could also undergo oxidation reactions. However, since we find that the optical properties of the films are stable during the electrochemical experiments (both in the LECs, Figure 4, and in the SECs experiments, Figure 5), it is unlikely that this is the dominant cause of irreversible hole injection. Finally, we consider that the injection efficiency for hole injection can be due to inefficient hole tunneling through the CdS/ZnS shells and any separation between the QDs. The valence band offset for hole tunneling through the shells is much larger than the conduction band offset for electron tunneling. In addition, the larger effective mass of holes will result in much slower hole tunneling.

Both electron and hole injections are required for LECs to exhibit EL. In polymer-based LECs, electron injection is typically less efficient than hole injection, causing the emissive intrinsic zone to be close to the cathode. In that case, the electrode-induced quenching limits the device performance. Similarly, inefficient hole injection in QLECs causes the emission zone to be close to the anode. Since we are not using any hole injection (*i.e*., electron blocking) layer, this could result in electron leak currents when electrons pass through the junction directly into the positive electrode. Thus, although the EDL facilitates some hole injection in QLECs, as evidenced by the observation of EL, boosting hole injection further is essential to improve the efficiency of QLECs, and in general, the use of EDLs as a tool for charge injection in QD devices.

## Conclusions

In conclusion, this proof-of-concept work highlights the possibility of employing QDs as the only active material in LECs, wherein they combine the roles of electrochemical doping, charge transfer, and electroluminescence. QLECs were fabricated without any charge injection and transport layers using highly photoluminescent ligand-exchanged CdSe/CdS/ZnS QDs. These devices have a much simpler device architecture than current QLEDs and QLECs. This facilitates the study of electron and hole injection and the formation of p- and n-doped regions in the devices.

The QLECs devices reproducibly show band-edge EL. The shift of EL spectra with changing voltage is minor, and a high color purity is maintained. Experimental and simulated *J*–*V* curves indicate that our devices work as LECs rather than diodes. The occurrence of electrochemical doping of the QDs in our devices has been experimentally confirmed by operando PL measurements. The external quantum efficiency of the proof-of-concept QLECs is so far limited to 0.024%, which is not comparable to the state-of-the-art LECs based on polymers.^64,65^ Spectroelectrochemical measurements demonstrate that electrochemical electron doping is efficient, but hole doping is nearly absent in these CdSe/CdS/ZnS QDs, significantly limiting the efficiency of the QLECs. Improvements in the efficiency and stability of hole injection are needed to enhance the device performance of QLECs to a level where they may become relevant for display and lighting applications.

## Experimental Section

### Materials

Indium nitrate hydrate (In­(NO_3_)_3_, 99.999%), poly­(ethylene oxide) (PEO, Mv ≈ 5 × 10^6^ g mol^–1^ and Mn ≈ 600 g mol^–1^), lithium trifluoromethanesulfonate (LiCF_3_SO_3_, 99.995%), lithium perchlorate (LiClO_4_, battery grade), ferrocenium hexafluorophosphate (FcPF_6_, 98%, BLDpharm), and anhydrous *N,N*-dimethylformamide (DMF), 99.8%, anhydrous acetonitrile (MeCN, 99.8%) were all purchased from Sigma-Aldrich unless otherwise stated and used as received. Aluminum (Al) pellets (99.99%) for thermal evaporation were purchased from the Kurt J. Lesker Company. Indium-doped tin oxide (ITO)/glass substrates (7–10 Ω/□) for QLECs and spectroelectrochemistry (SEC) measurements were purchased from MSE Supplies.

### Ligand Exchange

The ligand exchange of QDs was based on the method reported by Xiao et al.^55^ The ligand exchange solution was prepared by heating the mixture of In­(NO_3_)_3_ in DMF to 120 °C for 20 min with a concentration of 0.1 M. In a one-phase ligand exchange system, 1 mL of In­(NO_3_)_3_ solution (0.1 M) was added to 10 mL of QD solution (10 mg/mL). The mixture was vigorously stirred at room temperature until a precipitate was clearly observed. The mixture was purified twice by adding excess toluene and centrifuging at 1937 g. The resulting precipitate was dispersed in 1 mL DMF and filtered through PTFE syringe filters with a pore size of 0.2 μm. The QD solution was stored in a nitrogen-purged glovebox for future use. A two-phase ligand exchange system was selected for the illustration images in the inset of Figure 1a. In a 4 mL vial, 1.5 mL of 10 mg/mL QDs solution (in hexane) and 1.5 mL of 0.01 M In­(NO_3_)_3_ DMF solution were mixed and vigorously stirred until QDs were transferred from the hexane phase to the DMF phase.

### Device Fabrication and Characterization

The master solution was prepared by mixing 1 mL of QD solution (OD ≈ 1.2 at 633 nm in DMF), 0.5 mL of 10 mg/mL PEO solution (in DMF), and 0.1 mL of 10 mg/mL LiCF_3_SO_3_ solution (in DMF). It was stirred at 90 °C for 3 h. The patterned ITO/glass substrates were ultrasonically cleaned with acetone and isopropanol, followed by the ozone treatment. The QLECs with a structure of ITO/QD active layer/Al were fabricated by spin coating an active layer (∼100 nm) at 700 r.p.m. for 120 s onto the ITO/glass substrates, followed by drying at 4000 r.p.m. for 60 s. The films were subsequently annealed at 60 °C for 3 h. Afterward, Al electrodes (100 nm) were deposited using a thermal evaporation system through a shadow mask under a high vacuum of ∼1 × 10^–6^ mbar. The device area was 3 mm × 3 mm as defined by the overlapping area of the ITO and Al electrodes.

The current density–voltage measurements were performed by using a PGSTAT128N Autolab potentiostat. The emission from QLECs was measured by using a calibrated Si switchable gain photodetector (PDA100A2, Thorlabs). The luminance was calculated by assuming Lambertian emission. The electroluminescence spectra were recorded by using a fiber-coupled USB2000+ spectrometer (Ocean Optics). The operando photoluminescence measurement was performed by exciting the QLECs from the glass side and simultaneously recording the photoluminescence spectra during cyclic voltammetry, as is illustrated in Figure 4a. The excitation light source was a collimated laser diode with a wavelength of 405 nm and a power of 4.5 mW. The laser beam dimension was decreased by adding an iris (ID25Z/M, Thorlabs) between the laser and the device to ensure that QDs outside the actual area were not illuminated to contribute to the signals. The photoluminescence spectra were recorded simultaneously by using a fiber-coupled USB2000+ spectrometer (Ocean Optics). Due to the weak electroluminescence from our devices, the signal received from the device is mainly photoluminescence.

### SEC Measurement

The SEC experiments were performed using a PGSTAT128N Autolab potentiostat to regulate the potential and measure the current. A three-electrode electrochemical cell was used, consisting of a platinum (Pt) plate counter electrode, a Ag wire pseudoreference electrode (PRE), and a QDs/ITO/glass working electrode. The working electrode was prepared by drop-casting ligand-exchanged QD solution onto the ITO/glass substrate. The supporting electrolyte was 0.1 M LiClO_4_ PEO (600 g mol^–1^) or MeCN solution. All of the experiments were performed inside a nitrogen-purged glovebox. A scheme of the SEC measurement setup can be found in Figure S2. Cyclic voltammograms were obtained at a scan rate of 5 mV/s. The Ag PRE was calibrated with a ferrocene/ferrocenium redox couple. During the electrochemical doping, simultaneous differential absorption spectra and photoluminescence spectra were recorded on a fiber-coupled USB2000+ spectrometer (Ocean Optics). The white excitation light for the absorbance measurement was a DH-2000 deuterium halogen UV–vis-NIR light source (Ocean Optics).

### Simulations

The drift-diffusion simulator was based on the work by Van Reenen et al.^57^ The one-dimensional simulated space encompasses both electrodes and the active layer in between. The space is split into 250 individual cells, with the outermost cells representing the electrodes, and the cells in between representing the active layer. The active layer is modeled as a mixture of a semiconductor and an electrolyte, and the concentrations of electrons, holes, cations, and anions are tracked over time as a function of space. At time = 0, anions and cations are distributed equally over the active layer cells, and a small thermal population of both holes and electrons is present (concentrations are also constant over space). The concentrations of ions in and the ion current into the electrodes are always kept at zero. The simulator then starts iterating timesteps. During each time step, a midpoint method is used to solve the Poisson equation (Table S1) and to determine the spatial profile of the electrostatic potential for the next step. The boundary conditions for the Poisson equation are the potential values of the two electrodes, with the difference in these potentials being equal to the applied voltages at that time step. The applied voltage can either be constant or follow some program as a function of time (e.g., a linear scan). After the potential profile in space is determined, the movement of all carriers is calculated based on drift-diffusion equations (see Table S1 for all equations used in the simulation). Electron and hole concentrations in the cells adjacent to both electrodes are calculated by the Boltzmann approximation, assuming chemical equilibrium between the electrodes and the cells directly adjacent to them. Recombination of electrons and holes is governed by a second-order function. Table S2 shows the parameters employed in the simulation that were found to best match the experimental data and were used for the simulations shown in this work. Electron and hole currents are recorded by counting the number of electrons/holes that flow from the electrodes into the respective adjacent cells of the active layer.

## Supplementary Material



## Notes

Synthesis protocols and characterization of CdSe/CdS/ZnS QDs, formulas and parameters employed in the simulator, additional concentration and potential profiles in the simulated *J*–*V* curve of QLECs, illustration of SEC measurement setup, and SEC results obtained from 0.1 M LiClO_4_ MeCN electrolyte solution (PDF)
