# Supplementary material for: Potential of Electrochemical Charge Injection for Quantum Dot Light-Emitting Devices
Source: Chem Mater. 2025 Jun 2;37(12):4435–44. doi: 10.1021/acs.chemmater.5c00579 (PMC12199458; doi:10.1021/acs.chemmater.5c00579)
Supplement: Supplementary file 1 [file cm5c00579_si_001.pdf]

Supporting information for

The Potential of Electrochemical Charge Injection for Quantum Dot Light Emitting Devices

*Hua Chen‡, Reinout F. Ubbink‡, Rens A. Olsthoorn, Maarten Stam, Jesse 't Hoen, Tom J. Savenije and Arjan J. Houtepen**

Optoelectronic Materials Section, Faculty of Applied Sciences, Delft University of Technology, Van der Maasweg 9, 2629 HZ Delft, The Netherlands

Synthesis and Characterization of CdSe/CdS/ZnS QDs

Materials: Cadmium oxide (CdO, 99.99 %), oleic acid (OA, 90 %), 1-octadecene (ODE, 90 %), selenium powder (Se, 99.99 %), trioctylphosphine (TOP, 90 %), trioctylphosphine oxide (TOPO, 90 %), sulfur flake (S, 99.99 %), zinc acetate ($Zn(OAc)_2$, 99.99 %, anhydrous), oleylamine (OLAM, 80-90 %), octadecylamine (ODA, 99 %), deuterated dimethyl sulfoxide (DMSO-d_6, 99.9 atom %), deuterated chloroform ($CDCl_3$, 99.5 atom %, Cortecnet), rhodamine 6G (99 %) and anhydrous solvents (toluene, 99.8 %, methyl acetate, 99 % , methanol, 99.8 % , ethanol, 99.8 % and acetonitrile (MeCN), 99.8 %) were all purchased from Sigma-Aldrich unless otherwise stated and used as received, except for OA, ODE and OLAM, which were degassed in vacuo at 100 ˚C before being stored in the glovebox.

Precursor Preparation: 0.077 M Cadmium oleate (Cd-oleate) solution was synthesized by dissolving 0.367 g CdO in 3.68 g OA and 25.9 g ODE. The mixture was firstly degassed in vacuo at 110 ˚C for 1 h and then heated to 250 ˚C until a transparent solution was formed. Then it was cooled down to 110 ˚C and degassed again for 1 h. Afterwards, the reaction was cooled to room temperature and the Cd-oleate solution was stored in a nitrogen-purged glovebox for future use.

0.209 M zinc oleate (Zn-oleate) solution was synthesized by dissolving 0.367 g $Zn(OAc)_2$ in 1.13 g OA, 1.07 g OLAM and 5.523 g ODE in a 40 mL vial. Inside a nitrogen-purged glovebox, the vial was heated up to 130 ˚C until a transparent solution was formed and stored there for future use. OLAM stabilizes the Zn-oleate to prevent solidification at room temperature.[1]

0.75 M selenium precursor (Se-TOP) was prepared by heating up a mixture of 1.42 g Se, 7.5 g TOP and 11.9 g ODE to 60 ˚C until the complexation was completed.

0.5 M sulfur precursor (S-ODE) was prepared by heating up a mixture of 0.32 g S and 15.78 g ODE to 90 ˚C until the complexation was completed.

Synthesis of CdSe Core QDs: In a 100 mL three-neck round bottom flask, 3.2 g ODA and 1.11 g TOPO was heated up to 140 ˚C and degassed for 1.5 h. Afterwards, 5.2 g 0.75 M Se-TOP solution was added into the flask and the reaction was heated up to 300 ˚C. 4.9 g 0.077 M Cd-oleate solution was swiftly injected into the flask. The temperature was subsequently kept at 280 ˚C for 4 min. The reaction was quenched to 60 ˚C. To purify the CdSe core QDs, methyl acetate and methanol with a ratio of 5:1 was added to the reaction mixture, followed by centrifugation at 3354 g and redispersed in toluene. This purification procedure was repeated once. The corresponding diameter of CdSe core QDs was around 4 nm.

Synthesis of Core/shell/shell QDs: The shelling of CdSe core QDs was based on a method described in the work of Hanifi et al. with some modifications.[2] In this synthesis, S-ODE was used as sulfur source instead of thiols for the following ligand exchange. Using the methods below, CdSe/6CdS/2ZnS core/shell/shell QDs were synthesized and used in this work.

For the synthesis of 6 monolayers of CdS shell, 100 nmol CdSe core QDs in toluene, 4 mL 0.077 M Cd-oleate solution and 4 mL ODE were added to a 100 mL three-neck round bottom flask. The mixture was kept at 60 ˚C in vacuo for 1 h to remove toluene. Afterwards, the reaction mixture was heated up under nitrogen atmosphere. Upon the temperature reached 230 ˚C, 12 mL 0.04 M Cd-oleate solution and 12 mL 0.05 M S-ODE solution were dropwise

injected into the flask using two syringe pumps with a rate of 4 mL/h. During the injection, the temperature was kept at 310 ˚C. After the reaction was cooled down to room temperature, the crude product was purified by adding methyl acetate and methanol with a ratio of 5:1 and centrifuging at 8586 g. The precipitate was redispersed in toluene. The purification procedure was repeated once.

For the synthesis of 2 monolayers of ZnS shell, CdSe/CdS core/shell QDs synthesized above in toluene, 1.875 mL 0.209 M Zn-oleate solution and 3 mL ODE were added into a 100 mL three-neck round bottom flask. Toluene was removed by keeping the mixture at 60 ˚C in vacuo for 1 h. Afterwards, the mixture was heated up and when the temperature reached 230 ˚C, 12 mL 0.032 M Zn-oleate solution and 12 mL 0.064 M S-ODE solution were dropwise injection into the flask using two syringe pumps with a rate of 12 mL/h. The temperature was 280 ˚C during the injection of shelling precursors. CdSe/CdS/ZnS QDs were purified three times by adding methyl acetate:methanol (5:1) and centrifuging at 3354 g. The final precipitate was dispersed in toluene and stored in a nitrogen-purged glovebox for future use.

Characterization of QDs: The steady-state UV-vis absorption spectra were recorded using a PerkinElmer Lambda 365 spectrometer. The steady-state photoluminescence spectra were obtained using an Edinburgh Instruments FLS980 spectrofluorometer equipped with a 450 W Xenon lamp as the excitation source and double grating monochromators. PLQY of QDs were determined with respect to the rhodamine 6G reference dye in ethanol (PLQY 95%). The PLQY was calculated using the following equation:

$$PLQY = PLQY_{rhodamine\ 6G} \times \frac{I^{PL}_{QD\ solution}}{I^{PL}_{rhodamine\ 6G}} \times \frac{f_{rhodamine\ 6G}}{f_{QD\ solution}} \times \left(\frac{n_{QD\ solution}}{n_{ethanol}}\right)^2$$

Where I^{PL} is the integrated intensity of the photoluminescence spectra of either the QD solution or the rhodamine 6G solution, n is the refractive index of solvents used for either QD solutions or rhodamine 6G solution, f is the fraction of absorbed light of samples, calculated as f=1-10^(-OD), where OD is the optical density of the samples at the excitation wavelength (500 nm). All the samples were diluted until the OD at 500 nm was around 0.1.

Powder X-ray diffraction patterns were collected using a Bruker D8 Advance diffractometer (Cu Kα, λ=1.5418 Å). The samples were prepared by drop casting QD solutions onto the silicon substrates.

Solution nuclear magnetic resonance (NMR) spectra were recorded on an Agilent 400-MR DD2 equipped with a 5 mm ONE NMR probe and operating at 25 °C. 1H NMR (399.7 MHz) spectra were collected with a recycle delay of 1 s in $CDCl_3$ for oleate-capped QDs and DMSO-d6 for ligand exchanged QDs, respectively. Spectra were all calibrated with respect to the peak of tetramethylsilane (0 ppm).

Transmission electron microscopy (TEM) images were acquired using a JEOL JEM1400 transmission electron microscope which was operated at 120 kV. The TEM samples were prepared by drop casting QDs solution onto the carbon-coated copper TEM grids.

Table S1. List of formulas employed in the simulator.

Formula	Name and purpose	Explanation of parameters
$J_n = nq\mu_n \frac{dV}{dx} - kT\mu_n \frac{dn}{dx}$<br>$J_p = pq\mu_p \frac{dV}{dx} + kT\mu_p \frac{dp}{dx}$<br>$J_c = cq\mu_c \frac{dV}{dx} + kT\mu_c \frac{dc}{dx}$<br>$J_a = aq\mu_a \frac{dV}{dx} - kT\mu_a \frac{da}{dx}$	**Drift-diffusion equations**<br>Calculate the currents of electrons (n), holes (p), cations (c) and anions (a).	J: current density<br>q: elementary charge<br>V: electrostatic potential level vs vacuum<br>μ: carrier mobility
$\frac{d^2V}{dx^2} = \frac{q}{\varepsilon_0\varepsilon_r} (n - h + a - c)$	**1D Poisson equation**<br>Calculate the potential profile over the space of the simulation.	ε_0, ε_r: vacuum and relative electric permittivity.
$n = N_C * e^{\frac{E_f - E_C}{kT}}$<br>$p = N_V * e^{\frac{E_V - E_f}{kT}}$	**Boltzmann approximation**<br>Calculate the equilibrium concentration of electrons and holes at the interface between the electrodes and the active layer.	E: energy<br>N_C, N_V: effective density of states of the conduction, valence band<br>E_C, E_V: conduction, valence band level<br>E_F: Fermi level (= intrinsic Fermi level - electrostatic potential)
$R = k_R * n * p$	**Recombination**<br>Calculate the amount of recombination in each cell	k_R = recombination rate constant

Table S2. List of parameters used in this work. Parameters used in simulations were chosen to reflect those expected for QD-based active layers, then the mobilities were optimized to achieve a good fit with experimental results.

Parameter	Value (* = based on experimental data)
Active layer thickness	100 nm *
Number of lamella	250
Time step length	100 ns (1e-7 s)
Electron mobility	1.5e-12 $m^2V^{-1}s^{-1}$
Hole mobility	1.5e-12 $m^2V^{-1}s^{-1}$
Anion mobility	1e-15 $m^2V^{-1}s^{-1}$
Cation mobility	1.5e-12 $m^2V^{-1}s^{-1}$
Recombination rate constant	1e-11 m^3s^{-1}
Conduction band level	-3.9 eV vs vacuum (CdSe QDs) *
Valence band level	-5.9 eV vs vacuum (CdSe QDs) *
Work function of left electrode	-4.1 eV vs vacuum (Aluminum)
Work function of right electrode	-4.7 eV vs vacuum (ITO)
Effective density of states (holes and electrons)	1e26 m^{-3}
Ion concentration	5e25 m^{-3} (0.08 M) *
Temperature	300 K*
Relative permittivity of the active layer	4

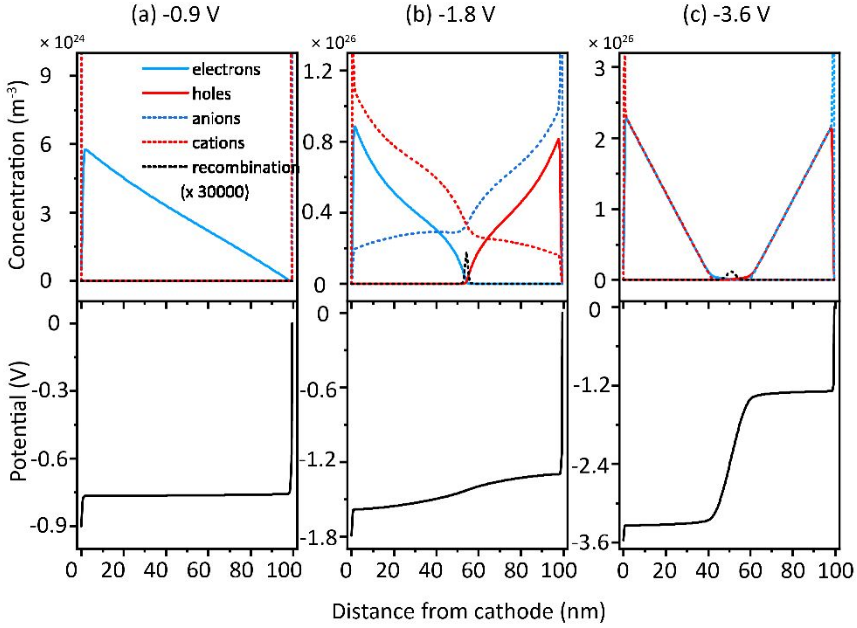

Figure S1 Concentration and potential profiles during the simulated J-V curve of the QLEC. a) below 1.7 V, EDL are formed at the electrodes, as evidenced by the potential drops at the side of the device. A small leak current of electrons occurs, but no electrochemical doping has taken place. b) between 1.7 and 2.7 V, the electrochemical doping process takes place. Electrons and holes are injected into the active layer, and separation of ions occurs to compensate for the added charge. An n-type doped region develops at the left of the device and a p-type doped region at the right. c) At -3.6 V, complete separation of cations and anions has taken place and the doped regions have completely developed. In the middle of the device a potential drop marks the position of the p-i-n junction.

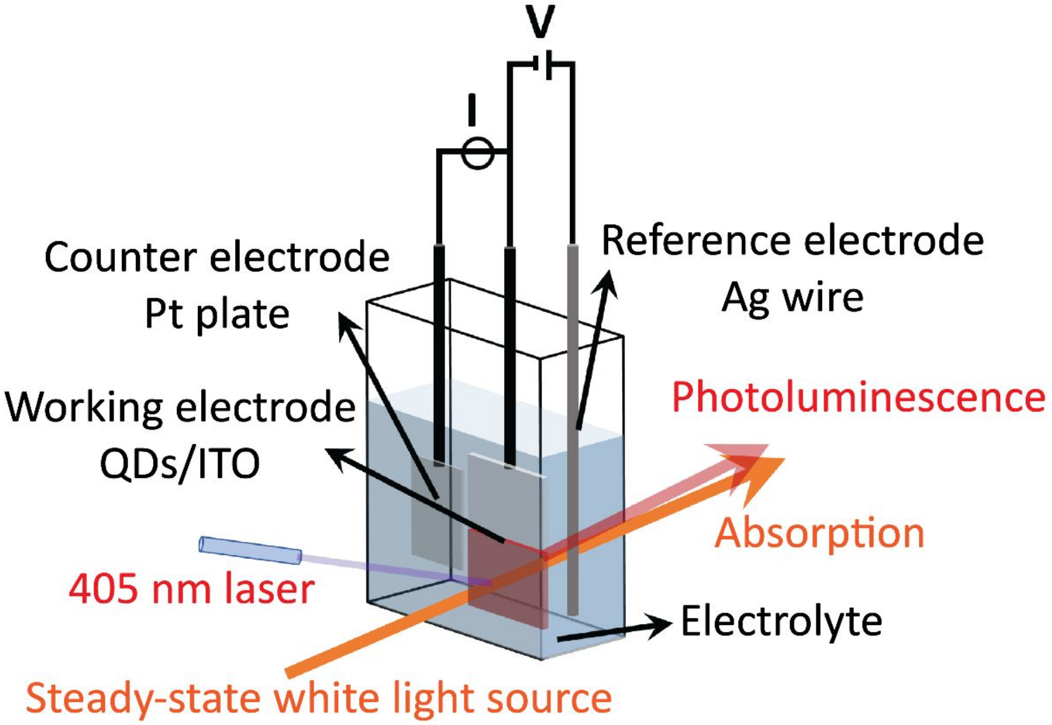

Figure S2. Illustration of spectroelectrochemistry measurement setup, including a Pt sheet as the counter electrode, a Ag wire as the reference electrode and a QD film on ITO substrate as the working electrode in the electrolyte solution. A laser with a wavelength of 405 nm and a steady-state white light source were used as the excitation light source for PL and absorption measurements.

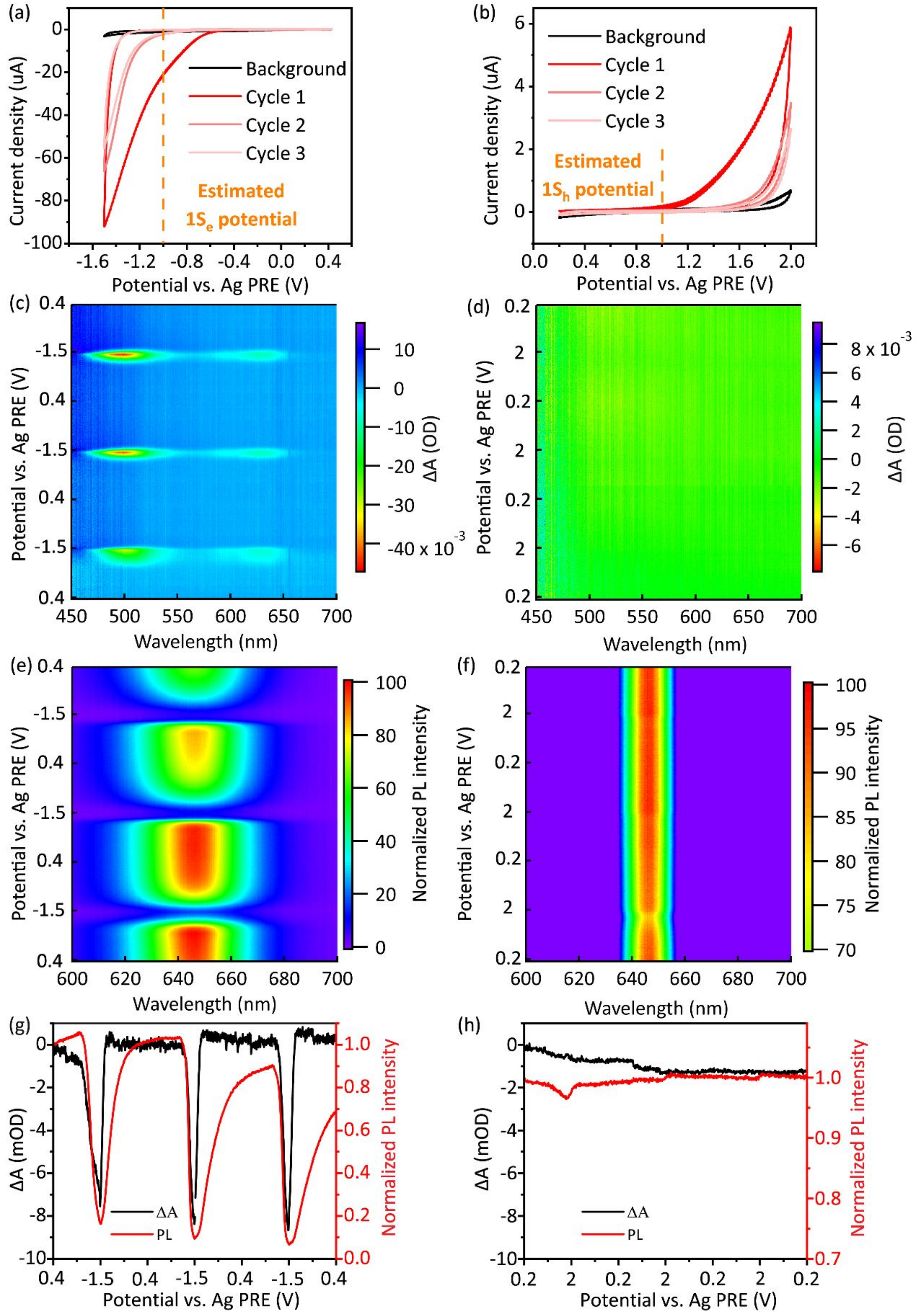

Figure S3. Cyclic voltammograms, 2D differential absorbance, 2D normalized PL and 1D plot of differential absorbance at CdSe 1S transition peak and PL peak maxima of QD films/ITO scanned a,c,e,g) negatively and b,d,f,h) positively in the 0.1 M $LiClO_4$ acetonitrile solution. The scan rate was 5 mV/s.

References

1. Geuchies, J. J.; Brynjarsson, B.; Grimaldi, G.; Gudjonsdottir, S.; van der Stam, W.; Evers, W. H.; Houtepen, A. J. Quantitative electrochemical control over optical gain in quantum-dot solids. *ACS Nano* 2021, *15* (1), 377–384.
2. Hanifi, D. A.; Bronstein, N. D.; Koscher, B. A.; Nett, Z.; Swabeck, J. K.; Takano, K.; Schwartzberg, A. M.; Maserati, L.; Vandewal, K.; van de Burgt, Y.; Salleo, A.; Alivisatos, A. P. Redefining near-unity luminescence in quantum dots with photothermal threshold quantum yield. *Science* 2019, *363* (6428), 1199–1202.
